# Hyper-Brain Networks Support Romantic Kissing in Humans

**DOI:** 10.1371/journal.pone.0112080

**Published:** 2014-11-06

**Authors:** Viktor Müller, Ulman Lindenberger

**Affiliations:** Center for Lifespan Psychology, Max Planck Institute for Human Development, Berlin, Germany; University of Namur, Belgium

## Abstract

Coordinated social interaction is associated with, and presumably dependent on, oscillatory couplings within and between brains, which, in turn, consist of an interplay across different frequencies. Here, we introduce a method of network construction based on the cross-frequency coupling (CFC) and examine whether coordinated social interaction is associated with CFC within and between brains. Specifically, we compare the electroencephalograms (EEG) of 15 heterosexual couples during romantic kissing to kissing one’s own hand, and to kissing one another while performing silent arithmetic. Using graph-theory methods, we identify theta–alpha hyper-brain networks, with alpha serving a cleaving or pacemaker function. Network strengths were higher and characteristic path lengths shorter when individuals were kissing each other than when they were kissing their own hand. In both partner-oriented kissing conditions, greater strength and shorter path length for 5-Hz oscillation nodes correlated reliably with greater partner-oriented kissing satisfaction. This correlation was especially strong for inter-brain connections in both partner-oriented kissing conditions but not during kissing one’s own hand. Kissing quality assessed after the kissing with silent arithmetic correlated reliably with intra-brain strength of 10-Hz oscillation nodes during both romantic kissing and kissing with silent arithmetic. We conclude that hyper-brain networks based on CFC may capture neural mechanisms that support interpersonally coordinated voluntary action and bonding behavior.

## Introduction

Brain activity and neural coupling during human social interaction have become a topic of scientific inquiry [1]–[6]. Prominent examples for activities requiring the close coordination of behavior in real time are performing music [7]–[10], singing [11], dancing [12], [13], collective sports [12]–[14], and bonding behaviors such as kissing [15]. Interpersonally coordinated behavior may reflect basic dispositions and needs [16], [17], and pleasure associated with such behavior may reinforce activities serving important evolutionary functions such as sexual reproduction and early mother–child interaction.

There is neurophysiological evidence that coordinated behavior is accompanied by synchronized brain activity [7]–[10], [18], [19] and oscillatory coupling of other biological functions, such as respiration and cardiac activity [11]. To investigate these phenomena, various synchronization or coupling measures have been proposed and used [7], [8], [11], [20]. Usually, when using time-frequency decompositions, brain networks are constructed and considered for specific frequencies [8]–[10], [18], [20]. However, based on conceptual considerations [21], [22], it seems important to also consider coupling *across* frequencies. For instance, large-scale theories of neural organization (e.g., [23]) and prior research [22], [24], [25] strongly suggest that cross-frequency coupling (CFC) plays a critical rule in neural information exchange. To overcome the limitations of single frequency representations, we extended the phase synchronization measures used in our earlier work [8], [11] to CFC. We then used these coupling indexes to construct hyper-brain networks that represent intra- and inter-brain synchronization within and across frequencies. Our novel approach allows researchers to represent the complex interplay among different frequencies in the context of a hyper-brain network (i.e., a network consisting of interacting nodes across two or more brains of interacting people). In comparison to all earlier approaches, where different brain sites (different electrodes in the case of the EEG) are defined as nodes, we considered single brain sites oscillating at different frequencies as different nodes by using a CFC measure. In other words, each node is a combination of an electrode location and of an oscillation frequency. Thus, the hyper-brain networks considered in this article consist of electrode-frequency nodes of two brains.

Measures derived from graph theory are increasingly being used in the neurosciences [26]–[33]. In modularity analyses, when using novel CFC approach, the same electrode can participate in different modules of the hyper-brain network dependent on the oscillation frequency. It is well known that single neurons and also brain areas can be involved in multiple overlapping cell assemblies [34]–[37]. Such assemblies oscillating synchronously at different frequencies provide an efficient basis for integrative processes in the brain [38]. CFC, allowing accurate timing between different oscillatory rhythms, may indicate integration or communication between different cell assemblies (cf. [39]–[41]). We assume that hyper-brain networks constructed by using CFC both within and between brains provide a more comprehensive representation of neural processes supporting interpersonal action coordination than hyper-brain networks that only consider couplings within particular frequency bands.

Accordingly, this study had two major aims: First, the methodological aim was to introduce a novel CFC approach for network construction that allows for the investigation of different oscillation frequencies in a common network. Second, the substantive goal was to investigate intra- and interbrain coupling during kissing, which we consider an ecologically valid evolved behavior. By simultaneously recording the EEG from two brains (e.g., EEG hyper-scanning), we compared CFC in romantic couples who engage in different varieties of kissing. We paid special attention to the way in which theta and alpha oscillation-nodes participate in hyper-brain *modules* and together constitute a theta-alpha subnetwork binding the brains of the kissing couple together. In particular, we show that CFC network properties during romantic kissing (RK), a two-person activity with intense reciprocal sensory and motor contact, but also during another partner-oriented kissing condition, in which the interaction partners are asked to perform a secondary, solitary activity while kissing each other (i.e., kissing while performing silent arithmetic, K-SA), are associated with elevated levels of intra- and inter-brain coupling and shorter path length than a solitary control condition without such contact – kissing one’s own hand (HK).

After the experiment, the participants were asked about partner-oriented kissing satisfaction as well as other partnership-related questions. We observed reliable correlations between network properties and subjectively assessed partner-oriented kissing satisfaction and self-reported kissing quality. These correlations point to the functional significance of theta and alpha oscillations in kissing couples. We complement our analyses with couplings between lips (assessed by EMG) and brains, again within and across individuals.

## Materials and Methods

### Participants

Twenty heterosexual couples who reported to be in love with each other, not earlier than 2 years ago (to achieve a homogeneous sample of romantic couples in love), participated in the study. Five couples were excluded from the data analysis because of recording artifacts. Analyses presented here are based on the remaining 15 couples. Participants were aged between 18 and 37 years with a mean age of 25.4 years (SD = 4.4). The Ethics Committee of Max Planck Institute for Human Development approved the study, and it was performed in accordance with the ethical standards laid down in the 1964 Declaration of Helsinki. All participants volunteered for this experiment and gave their written informed consent prior to their inclusion in the study.

### Procedure

Each couple participated in seven solitary and eight partner-oriented or common-percept experimental task conditions lasting for 3–4 minutes each. The seven solitary task conditions were followed by the eight partner-oriented or common-percept task conditions. Here we report the data from three different kissing conditions: HK, RK, and K-SA. To safeguard the ecological validity of the romantic kissing condition, RK always preceded the K-SA condition. While kissing, participants were asked to keep their eyes closed and to either kiss their own hand (solitary condition) or kiss each other’s lips (partner-oriented condition with and without additional task). In the task condition (K-SA), the participants were each asked to subtract 7 repeatedly from the remaining amount, beginning with the number 1,000 during kissing. At the end of this kissing condition, participants were asked to provide their subtraction result.

### Psychological assessment

We assessed the partners’ feelings during the test session, partnership and kissing satisfaction as well as the quality of the kissing. Most items were based on a 5-point rating scale ranging from 1 (not at all) to 5 (very much). The items are summarized in Table 1 and Table S1. The assessment was carried out during and after the EEG session. Kissing quality was assessed immediately after the RK and K-SA sessions, correspondingly. The other items were assessed after the entire EEG session.

**Table 1:** Psychological assessment of partner-oriented kissing satisfaction and immediate kissing quality during the experiment.

Items	female		male	
**Partner-oriented kissing satisfaction**				
How well do you harmonize with your partner while kissing?	4.3 (0.9)		4.5 (0.6)	
How well does your partner kiss?	4.7 (0.5)		4.5 (0.6)	
How important is kissing in your relationship?	4.3 (0.6)		4.0 (0.9)	
**Immediate kissing quality during experiment**				
	**RK**	**K-SA**	**RK**	**K-SA**
How much did your kissing today resemble the way you normally kiss?	3.6 (0.8)	2.3 (1.1)	3.3 (1.0)	2.0 (0.8)
How successful was your kissing during the experiment?	4.1 (0.7)	2.9 (0.8)	3.6 (1.0)	2.7 (1.0)
How intense was your kissing?	4.1 (0.8)	2.9 (1.1)	3.5 (1.0)	2.1 (0.9)

RK = romantic kissing, K-SA = kissing while performing silent arithmetic.

### EEG data acquisition and preprocessing

EEG measurement took place in an acoustically and electromagnetically shielded cabin. Separate amplifiers with separate grounds were used for each individual, optically coupled to a computer. Under all task conditions, the EEG was simultaneously recorded from both participants using two electrode caps with 64 Ag/AgCl electrodes placed according to the international 10–10 system, with the reference electrode at the right mastoid. Vertical and horizontal electrooculograms (EOGs) were recorded to control for eye blinks and eye movements. Additionally, a bipolar lip EMG was obtained from two EMG electrodes placed over the orbicularis oris muscle. The sampling rate was 5000 Hz. Recorded frequency bands ranged from 0.01 to 1000 Hz. EEG recordings were re-referenced to an average of the left and right mastoid separately for each subject, resampled at 1000 Hz, and filtered with a band pass ranging from 1 to 70 Hz. Eye-movement correction was accomplished by independent component analysis [42]. Using the lip EMG channels, which were filtered with a high-pass filter of 4 Hz, we manually pasted markers on the kiss onset. Spontaneous EEG activity was then divided into epochs of 3 sec, starting 500 ms before the kiss onset and ending 2500 ms after it. Artifacts from head and body movements were rejected by visual inspection, after an artifact rejection based on a gradient (a maximum admissible voltage step of 50 µV), and a difference criterion (a maximum admissible absolute difference of 200 µV between two values in a segment) had not rendered satisfactory results. Epochs that were artifact-free in both participants were selected for further analysis. To reduce the amount of data and to overcome the problem of volume conduction between neighboring electrodes, we selected 21 electrodes based on the 10–20 system (Fp1, Fpz, Fp2, F7, F3, Fz, F4, F8, T7, C3, Cz, C4, T8, P7, P3, Pz, P4, P8, O1, Oz, and O2). These electrodes are distributed across the entire cortex so that the information of the remaining electrodes would be rather redundant.

In the EEG and lip EMG data, power spectra were calculated using the Fast Fourier Transform (FFT) with the Hanning window. In the EEG spectra, we then determined the individual alpha peak frequency and the spectral power in the low (6.8–9.7 Hz) and high (9.7–12.6 Hz) alpha frequency bands separately for each kissing condition. In the lip EMG spectra, we determined average power at the individual maximum (±10 Hz) and at 60 Hz (±10 Hz). Power values were normalized using a logarithmic transformation.

### Description of phase synchronization or coupling measures

To investigate phase coupling in a directed and frequency-resolved manner, we first applied an analytic or complex-valued Morlet wavelet transform to compute the instantaneous phase in the frequency range from 0 to 100 Hz in 0.5-Hz steps. The complex mother Morlet wavelet, also called Gabor wavelet, has a Gaussian shape around its central frequency *f*:
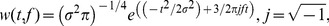
(1)in which σ is the standard deviation of the Gaussian envelope of the mother wavelet. The wavelet coefficients were calculated with a time step of 5, leading to a time resolution of 5 ms and frequency resolution of 0.5 Hz. In order to identify the phase relations within and between any two channels or frequencies, the instantaneous phase difference was then computed from the wavelet coefficients for all possible electrode and frequency pairs within and between the brains. On the basis of instantaneous phases for two signals (*X* and *Y*) given as: Φ*_X_*(*f_m_,t*) = arg[*φ_X_*(*f_m_,t*)] and Φ*_Y_*(*f_n_,t*) = arg[*φ_Y_*(*f_n_,t*)], correspondingly, the *n:m* phase synchronization between two oscillations at the frequencies *f_m_* and *f_n_* were determined. The generalized phase difference (ΔΦ) according to *n·f_m_* = *m·f_n_* was calculated by:



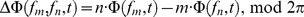
(2)The *n:m* phase synchronization index (*PSI*) was then defined by:
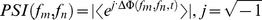
(3)where <•> denotes the averaging across *time*. In the case of within-frequency coupling (WFC) with *f_m_* = *f_n_*, *PSI* is calculated in the same way by setting *m* = *n* = 1. During calculation of the *PSI*, we not only determined the mean direction or the length of the vector but also the angle of this vector (*θ*) in complex space:



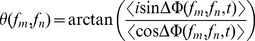
(4)To determine the directed cross-frequency coupling, we used the *adaptive Integrative Coupling Index* (*aICI*) in this work. In contrast to our earlier studies [8], [11], we used an *adaptive* algorithm, which allowed us to calculate coupling depending on the angle of phase differences determined in a given time window. In other words, *aICI* no longer reflects *in-phase* synchronization, where the angle of phase differences is close to zero, but is suitable for the determination of phase coupling at any chosen or previously determined phase angles (e.g., *θ*).

**Figure 1:**
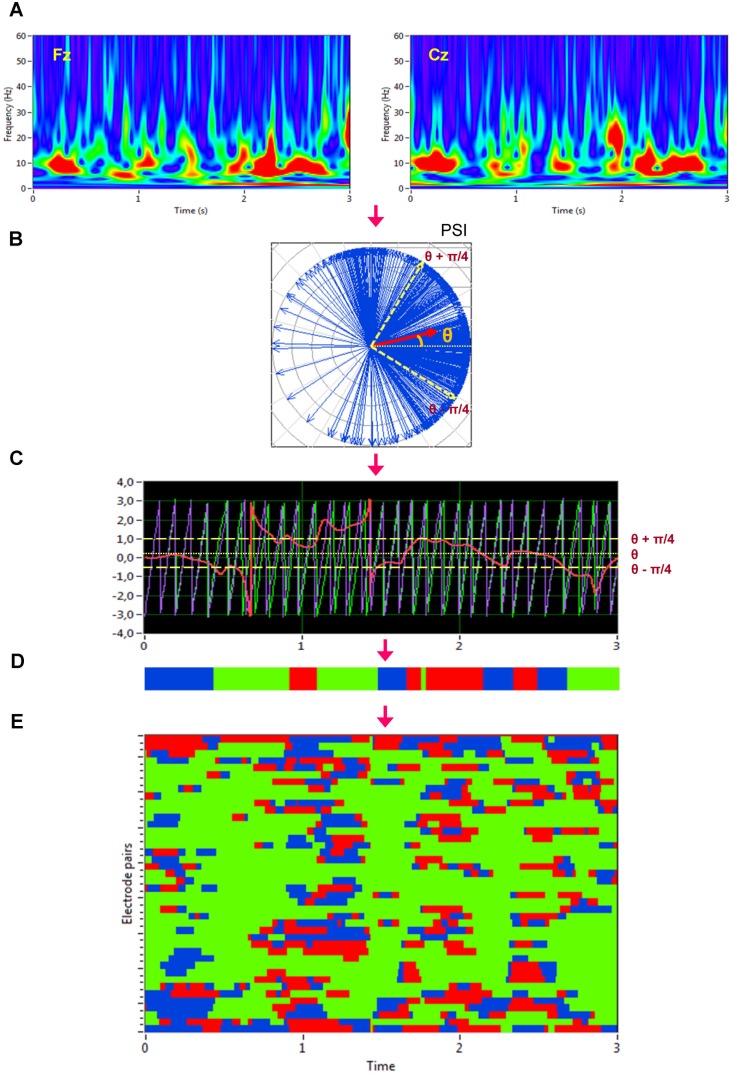
Calculation of the adaptive Integrative Coupling Index *(aICI).* **A:** Complex Morlet wavelet transformation of signals from two channels (Fz and Cz) in the time-frequency domain. **B:** The phase difference is depicted in the form of the vectors in complex space, where the blue arrows reflect single phase angles and the red arrow represents the mean vector of the angular dispersions (its length displays the *PSI* measure); θ is angle of this mean vector. Boundaries for calculation of *aICI* (θ−π/4 and θ+π/4) are indicated by the yellow dashed arrows. **C:** Time course of instantaneous phases from two channels (Fz and Cz) at *f_i_* = 10 Hz and their phase difference (Fz = violet curve; Cz = green curve; Fz–Cz = red curve). Angle of the mean vector *θ* and boundaries for calculation of *aICI* (*θ*−π/4 and *θ*+π/4) are indicated by yellow dotted and dashed lines. **D:** Coding of the phase difference of two signals, S1 (e.g., Fz) and S2 (e.g., Cz), at a given frequency (θ−π/4<S1–S2< θ: blue stripes; θ<S1–S2<θ+π/4: red stripes; S1–S2<−π/4 or S1–S2>+π/4: green stripes = nonsynchronization). **E:** Pair-wise synchronization pattern of all possible electrode pairs with Fz as a reference electrode. Each line represents one pair of channels.



Given the estimates of the phase difference between two signals, it is possible to ascertain how long the phase difference remains stable in defined phase angle boundaries by counting the number of points that are phase-locked at a defined time window. We slightly modified the procedure described in Müller and colleagues [8] in that we defined phase angle boundaries not related to phase zero but to the phase angle *θ*. The further procedure was similar, as depicted in Figure 1. After the complex wavelet transform of the signals (Fig. 1A) and determination of *PSI* and *θ* (Fig. 1B), we divided the range between *θ*−π/4 and *θ*+π/4 into two ranges and distinguished between positive and negative deviations from phase *θ* (Fig. 1C). As shown in Figure 1D, we marked negative deviations in the range between *θ*−π/4 and *θ* in blue (coded with “−1”) and the positive deviations in the range between *θ* and *θ*+π/4 in red (coded with “+1”). Phase difference values beyond these range were marked with green (coded with “0”) and represent non-synchronization. In the case of two channels, *X* (e.g., Fz) and *Y* (e.g., Cz), a blue stripe in the diagram would mean that the phase of channel *Y* precedes the phase of channel *X* and a red stripe would mean that the phase of channel *X* precedes the phase of channel *Y*. We then counted the number of data points that are phase-locked separately in each of these two ranges. Before counting, successive points in the defined range (between *θ*−π/4 and *θ*+π/4) with a time interval shorter than a period of the corresponding oscillation at the given frequency (*T_i_* = 1/*f_i_*) were discarded from the analysis. In the case of CFC, the lower frequency was considered. This cleaning procedure effectively eliminated instances of accidental synchronization. Figure 1E represents synchronization pattern of several electrode pairs after this cleaning procedure. On the basis of the counting described above, we obtained several synchronization indices: (1) the Positive Coupling Index, *PCI*, or the relative number of phase-locked points in the positive range (between *θ* and *θ*+π/4); (2) the Negative Coupling Index, *NCI*, or the relative number of phase-locked points in the negative range (between *θ*−π/4 and *θ*); (3) the Absolute Coupling Index, *ACI*, or the relative number of phase-locked points in the positive and negative range (i.e., between *θ*−π/4 and *θ*+π/4) indicating absolute synchronization; (4) the adaptive Integrative Coupling Index, *aICI*, calculated by the formulae:

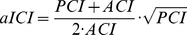
(5)The *aICI* measure ranges from 0 to 1. It is an asymmetric measure (i.e., *aICI*_AB_≠*aICI*_BA_) expressing both common (absolute) and “positive” contributions to phase synchronization.

Phase differences and corresponding phase synchronization measures were determined for six different frequencies of interest (FOI): 5, 10, 20, 30, 40, and 60 Hz, and all corresponding combinations between them, reflecting different frequency relations, such as 1∶2, 1∶3, 1∶4, 1∶6, 1∶8, 1∶12, 2∶3, and 3∶4. In addition to the EEG-EEG coupling, we also determined lip–lip EMG and lip EMG–EEG coupling. In the case of lip EMG, only prominent 60-Hz oscillations were used. We restrict the description of our study results to the *aICI* measure, which is most informative due to its directionality.

### Graph-theoretical approach (GTA)

#### Network construction

The coupling measures determined as described above were used to construct a connectivity matrix or a graph determining the network properties. In comparison to all earlier approaches, where different brain sites (different electrodes in the case of the EEG) were defined as nodes in such a graph, we were able to define single brain sites oscillating at different frequencies as different nodes by using a CFC measure. This means that the same electrode site at the six FOI (5, 10, 20, 30, 40, and 60 Hz) represents six different nodes that communicate with other nodes at the same or different frequencies. In other words, each node is a combination of spatial representation (electrode location) and of the oscillation frequency. The structure of such a graph is represented in Figure S1. The advantages of a network architecture allowing for CFC are: (1) not only connections between but also within the brain areas can be captured; (2) different brain areas can communicate with each other at multiple frequencies. In addition to the 21 EEG electrodes of each of the two kissing partners at six different frequencies, two EMG lip responses of female and male partners at the prominent frequency of 60 Hz were used for the construction of our hyper-brain networks. There were 254 nodes altogether (2×21×6+2 = 254) in each network.

**Figure 2:**
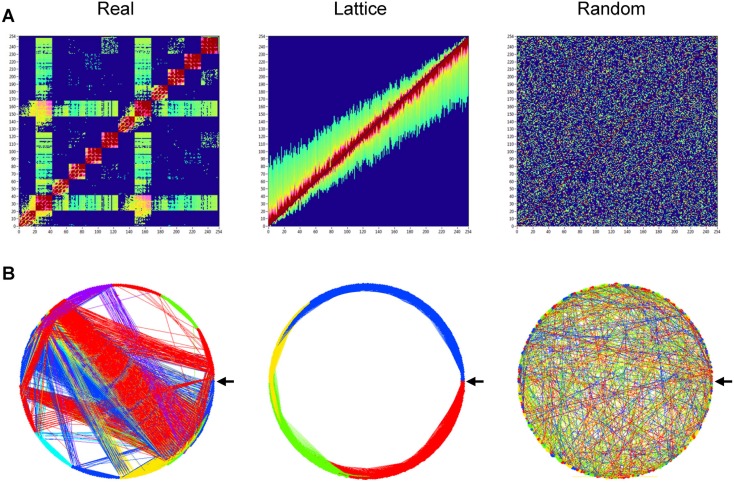
Representation of real, lattice, and random networks. **A:** Coupling *(aICI)* matrices covering within-frequency coupling (WFC) and cross-frequency coupling (CFC) between the 254 nodes of the network. In the real hyper-brain network (left), the nodes are organized by electrode location (Fp1, Fpz, Fp2, F7, F3, …, O2), oscillation frequency (5, 10, 20, 30, 40, and 60 Hz), and brains (female, male); the last two nodes are lip EMG channels oscillating at 60 Hz for a female and a male, correspondingly. The lattice network (middle) was configured by the randomization of the edges in the real network and consecutive redistribution in such a way that the strongest edges lay close to the main diagonal. The random network (right) was configured by the randomization of the edges only. The lattice and random networks were reconstructed in such a way that they have the same number of nodes and edges as the initial real network, but are characterized by ring (lattice) or random network topology. **B:** The same networks as in **A**, represented in the form of a circle, where the nodes are in clockwise order beginning with 0°, marked with arrow. Note: more information about network construction can be found in Figure S2.

To investigate the network topology of the real networks, we also constructed regular (lattice) and random networks that have the same number of nodes and mean degree as our real networks. For these purposes, we randomized the edges in the real network to achieve a random network with the same number of nodes and edges. Lattice networks were configured like random networks, but in addition edges were redistributed after an initial random permutation such that they lay close to the main diagonal with increasing order of their weights. For these purposes, each column in the adjacency matrix was split into two parts around the diagonal. Further, all edges in these two parts were redistributed in increasing order, and then merged again into the column. Lattice networks reconstructed in such a way have the same number of nodes and edges as the initial real network but are characterized by ring or lattice topology incorporating nearest-neighbor connectivity [43]. These network reconstructions for random and regular networks were carried out 10 times for each individual network. Average network topology was then determined for these repeated reconstructions. Real and correspondingly reconstructed networks are displayed in Figure 2 for comparison. For this representation, grand averages of connectivity values were used.

#### Threshold determination

Before determining the network properties by means of the GTA in a next step, the threshold of the synchronization or coupling measures had to be calculated. For this purpose we generated surrogate data by (a) computing the amplitude and phase spectrum of a real signal using a Fourier transformation; (b) phase shuffling, whereby the phase values of the original spectrum are used in random order and the sorted values of the surrogate sequence are replaced by the corresponding sorted values of the reference sequence; and (c) inverse Fourier transformation back to the time domain. In this way, the real and the surrogate data retain the same power spectrum but a different time course. Surrogate data computed in such a way for all epochs at all considered channels were then used for the calculation of the corresponding synchronization measures. Thereafter, we applied a bootstrapping procedure with 1,000 resamples of the coupling measures gained from the surrogate data set and determined the threshold as the bootstrapping mean plus the confidence interval at a significance level of p≤0.0001 separately for each frequency combination. Only coupling values greater than these threshold values were considered for network construction and further analyses.

In general, the choice of a threshold plays an important and nontrivial role in network construction, but is necessarily always arbitrary. In our case, the use of CFC makes this problem even more complex. To overcome this, at least partly, we (1) used different threshold values for different FOI and their combinations, as they were determined using the surrogate data procedure, and (2) applied a range of thresholds by the multiplication of initial threshold values by 10 different equidistant multiplication factors from 1.0 to 1.045 with a lag of 0.005. Thresholds determined in this way were used to construct different graphs with sparse connections covering a sparsity-range between 16% and 43% of the strongest edges preserved. Thus, we could compare the topological network properties at different sparsity or costs levels. As we were interested in coupling strengths, we used weighted networks at different sparsity levels.

### Network metrics

#### Degrees and strengths

As *aICI* is a directed measure, we obtained the node in- and out-degrees in the network, in which the in-degree is the sum of all incoming connections of the node *i* 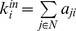, and the out-degree is the sum of all outgoing connections 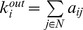. To calculate strengths, we then replaced the sum of links by the sum of weights, 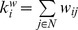, and calculated in- and out-strength, respectively. Thus, the strength can be considered as the weighted degree [32]. For statistical evaluation, we determined out-strengths for each of the nodes of the whole hyper-brain network of each kissing couple. Additionally, we determined the within-brain out-strength for each of the kissing partners, and then calculated the between-brain out-strength by subtracting the within-brain out-strength from the hyper-brain out-strength.

#### Clustering coefficient (CC) and characteristic path length (CPL)

If the nearest neighbors of a node are also directly connected to each other, they form a cluster. For an individual node, the clustering coefficient (*CC*) is defined as the proportion of the existing number of connections to the total number of possible connections. In the case of a weighted directed graph the mean *CC* is calculated by the formula [44]:
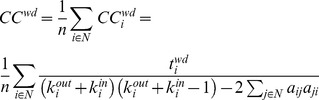
(6)


with 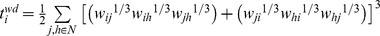 being the number of weighted directed triangles around a node *i*.

Another important measure is the characteristic path length (*CPL*). In the case of an unweighted graph, the shortest path length or distance *d_i,j_* between two nodes *i* and *j* is the minimal number of edges that have to be passed to go from *i* to *j*. This is also called the geodesic path between the nodes *i* and *j*. The *CLP* of a graph is the mean of the path lengths between all possible pairs of vertices [45]:
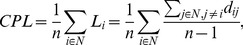
(7)where *L_i_* = *CPL_i_* is the average distance or average shortest path length between node *i* and all other nodes. In the case of a weighted and directed graph, the weight and direction of the links are considered.

#### Global (E_glob_) and local (E_loc_) efficiency

Global efficiency *(E_glob_)* is defined as the average inverse shortest path length and is calculated by [46]:
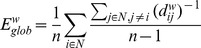
(8)


Like *CPL, E_glob_* is a measure of the integration of a network, but whereas *CPL* is primarily influenced by long paths, *E_glob_* is primarily influenced by short ones. Calculating *E_glob_* is advantageous over distance in disconnected networks: The efficiency between disconnected pairs of nodes is set to zero (the inverse of infinity).

Local efficiency *(E_loc_)* is similar to the *CC* and is calculated as the harmonic mean of neighbor-neighbor distances [46]:
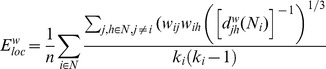
(9)Like *CC*, *E_loc_* is a measure of the segregation of a network, indicating efficiency of information transfer in the immediate neighborhood of each node and showing how fault-tolerant the system is.

#### Small-worldness

To investigate the *small-world* (SW) properties of a network it has become common to compare its clustering coefficient and characteristic path length to those of regular lattices and random graphs. At least two specific properties of small-world network (SWN) related to control networks (random and lattice) are significant: (1) The *CC* of the SWN (*CC_SWN_*) is much higher than that of random networks (*CC_SWN_*>>*CC_rand_*), but the CPL of the SWN (*CPL_SWN_*) is only slightly higher than that of the random network (*CPL_SWN_*≥*CPL_rand_*), and (2) the *CC* of the SWN is lower than that of lattice networks (*CC_SWN_*≤*CC_latt_*), but the *CPL* of the SWN is much lower than that of the lattice network (*CPL_SWN_*<<*CPL_latt_*). Specific quantitative SW metrics were developed in addition to these main graph metrics. Foremost, the so-called SW coefficient *σ*, is related to the main metrics of a random graph (*CC_rand_* and *CPL_rand_*) and is determined on the basis of two ratios 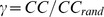 and 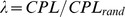 [47]:
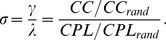
(10)


The SW coefficient *σ* has been used in numerous networks showing SW properties and has been found to be greater than 1 in the SWN (*σ*>1).

The second SW metric was defined by comparing the *CC* of the network of interest to that of an equivalent lattice network and comparing the *CPL* of the network to that of an equivalent random network [48]:
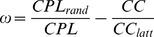
(11)


This metric normally ranges between −1 and +1 and is close to zero for SWN (CPL*_SWN_*≈CPL*_rand_* and CC*_SWN_*≈CPL*_latt_*). In addition, positive values of *ω* indicate a graph with more random characteristics (CPL*_SWN_*≈CPL*_rand_* and CC*_SWN_*<<CPL*_latt_*), while negative values indicate a graph with more regular (lattice-like) characteristics (CPL*_SWN_*>>CPL*_rand_* and CC*_SWN_*≈CPL*_latt_*). The clear advantage of the *ω* metric as compared to *σ* is the possibility to define the extent to which the network of interest is like its lattice or random equivalents [48].

#### Community structures and definition of node roles within the brain networks

To further investigate the topological properties of the hyper-brain networks, community structures as well as indices of modularity (*M*), the within-module degree (*Z_i_*) and the participation coefficient (*P_i_*) were determined (cf. [32]). For this calculation, the modularity optimization method for directed networks [49] as implemented in the Brain Connectivity Toolbox [32] was used (for limitations regarding this method, see [50]). The optimal community structure is a subdivision of the network into non-overlapping groups of nodes in a way that maximizes the number of within-module edges, and minimizes the number of between-module edges. The modularity (*M*) is a statistic that quantifies the degree to which the network may be subdivided into such clearly delineated groups or modules. It is given for directed networks by the formula [49]:
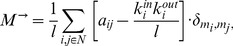
(12)where 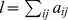 is the number of edges in the graph, and 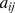 is defined to be 1 if there is an edge from *j* to *i* and zero otherwise, 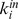 and 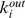 are the in- and out-degrees of the node *i*, and 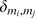 is the Kronecker delta. High modularity values indicate strong separation of the nodes into modules. *M* = 0 if nodes are placed at random into modules or if all nodes are in the same cluster [49]. To test the modularity of the empirically observed networks, we compared them to the modularity distribution (N = 100) of random networks, that is, to simulated networks with the same number of nodes and edges as the original network [51].

The within-module degree *Z_i_* indicates how well node *i* is connected to other nodes within the module *m_i_*. It is determined by [52]:
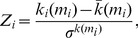
(13)where *k_i_*(*m_i_*) is the within-module degree of node *i* (the number of links between *i* and all other nodes in *m_i_*), 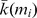 and 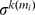 are the mean and standard deviation of the within-module degree distribution of *m_i_*. The within-module degree *Z_i_* is zero if all the nodes of the module have the same number of edges (e.g., if all the nodes within the module are fully interconnected with each other); otherwise it has negative or positive values depending on the number of links at the different nodes.

The participation coefficient *P_i_* describes how well the nodal connections are distributed across different modules [52]:
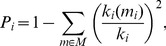
(14)where *M* is the set of modules, *k_i_(m_i_)* is the number of links between node *i* and all other nodes in module *m_i_*, and *k_i_* is the total degree of node *i* in the network. Correspondingly, *P_i_* of a node *i* is close to 1 if its links are uniformly distributed among all the modules, and zero if all of its links lie within its own module. *Z_i_*- and *P_i_*-values form a so-called *Z-P* parameter space and are characteristic for the different roles of the nodes in the network [52].

The community structures and corresponding measures (*M*, *Z_i_*, and *P_i_*) were determined for the common networks of the kissing couples for each kissing condition separately.

### Statistical analyses

Partner-oriented kissing satisfaction was analyzed using a two-way repeated measures ANOVA with a between-subject factor Sex (female vs. male) and a within-subject factor Items (3 Items, indicated in Table 1). Immediate kissing quality was assessed separately for RK and K-SA conditions and analyzed using a three-way repeated measures ANOVA, Sex×Items×Kissing (RK vs. K-SA). Alpha peak frequency was analyzed using a four-way repeated measures ANOVA with a between-subject factor Sex and three within-subject factors Kissing (RK, K-SA, and HK), Anterior-Posterior (frontal, central, parietal, and occipital), and Left-Right. Spectral power in the low and high alpha frequency band was subjected to a five-way repeated measures ANOVA with a between-subject factor Sex and four within-subject factors Kissing, Alpha Band, Anterior-Posterior, and Left-Right. Lip EMG at the individual maximum (±10 Hz) and at 60 Hz (±10 Hz) were analyzed using a two-way repeated measures ANOVA with a between-subject factor Sex and a within-subject factor Kissing (RK, K-SA, and HK). For statistical analyses of network properties, individual electrodes were collapsed into three regions along the anterior-posterior axis (frontal, central, and parieto-occipital) for each FOI separately. Strengths were statistically evaluated for hyper-brain networks and separately for within- and between-brain connections. Strengths, *CC* and *CPL* were analyzed using a four-way repeated measures ANOVA with a between-subject factor Sex (female vs. male) and three within-subject factors Frequency (5, 10, 20, 30, 40, and 60 Hz), Site (frontal, central, and parietal), and Kissing (RK, K-SA, and HK). Greenhouse-Geisser epsilons were used in all ANOVAs for non-sphericity correction when necessary. The Scheffe test was employed for the *post-hoc* testing of kissing condition differences.

To assess correlations between kissing satisfaction and quality and electrophysiological data, Pearson product correlations were computed between the averaged scores of partner-oriented kissing satisfaction or kissing quality and the network or other EEG measures. The difference between the correlation coefficients was tested using Steiger’s procedure [53].

## Results

### Psychological assessment

Table 1 summarizes psychologically assessed data (means and standard deviations) of partner-oriented kissing satisfaction and kissing quality during the experiment in the female and male partners. Further results of the psychological assessment can be found in the Table S1. Immediate kissing quality was assessed separately for RK and K-SA conditions. A two-way repeated measures ANOVA Sex×Items for partner-oriented kissing satisfaction revealed only significant item differences (F(2,56) = 7.30, P = 0.003, η^2^ = 0.21). A three-way repeated measures ANOVA Sex×Items×Kissing for immediate kissing quality revealed significant main effects of all three factors: Sex (F(1,28) = 5.83, P = 0.023, η^2^ = 0.17), Items (F(2,56) = 8.62, P = 0.001, η^2^ = 0.24), and Kissing (F(1,28) = 29.90, P<0.0001, η^2^ = 0.52), but no significant interactions. As shown in Table 1, women generally reported higher immediate kissing quality during the experiment than men, and kissing quality was rated higher for RK than for K-SA.

### EEG and lip EMG spectral power analyses

First, we determined alpha peak frequency and spectral power in low and high alpha frequency bands in each of the female and male partners during the three different kissing conditions. The alpha peak frequency differed by kissing condition (F(2,56) = 3.77, P = 0.029, η^2^ = 0.12). A posthoc Scheffe test showed significant differences between K-SA and HK (K-SA>HK: Mean Diff.  = 0.217, Crit. Diff.  = 0.205, P<0.05). Spectral power in the low and high alpha frequency bands varied as a function of the kissing condition, as indicated by significant interactions, Kissing×Alpha Band (F(2,56) = 3.62, P = 0.041, η^2^ = 0.12) and Kissing×Alpha Band×Anterior-Posterior (F(6,168) = 3.00, P = 0.025, η^2^ = 0.10), and as a function of Sex (Sex×Alpha Band×Anterior-Posterior, F(3,84) = 8.33, P = 0.003, η^2^ = 0.23 and ×Alpha Band×Left-Right, F(2,56) = 4.59, P = 0.025, η^2^ = 0.14). In general, spectral power was highest during RK and lowest during K-SA in the low alpha frequency band, and lowest during HK in the high alpha frequency band, especially at parieto-occipital regions. The spectral power in the low alpha frequency band was higher in men than in women, especially at parieto-occipital sites, and higher in women than in men in the high alpha frequency band at occipital and frontal regions.

Second, we determined the average power of lip EMG at the individual maximum (±10 Hz) and at 60 Hz (±10 Hz). Lip EMG oscillations at 60 Hz were used together with EEG channels for the construction of hyper-brain networks (see Methods). A two-way repeated-measures ANOVA (Sex×Kissing) revealed only a significant main effect of Sex for average spectral power at the individual maximum, F(1,28) = 4.6, P<0.05, η^2^ = 0.14, and a marginally significant main effect of Sex for average spectral power across 60 Hz, F(1,28) = 3.9, P = 0.058, η^2^ = 0.12, indicating a higher spectral lip EMG power in women than in men. There were no significant differences in the frequency of the maximum amplitude.

### Network analyses

As described in Methods and displayed in Figure S1, we constructed our hyper-brain networks using CFC measures between 21 EEG electrodes of each of the two kissing partners at six different frequencies and two EMG lip responses of female and male partners at the prominent frequency of 60 Hz. There were 254 nodes altogether (2×21×6+2 = 254) in each network (see Figure S1 for details). To investigate the SW properties of the real networks, we constructed two different control networks: regular (lattice) and random networks containing the same number of nodes and edges. Figure 2 displays network structures for real, lattice, and random networks using grand average coupling values. As expected, the lattice network showed a regular structure with high connectivity between neighbors, while random networks had a random structure with equally distributed short- and long-range connections. Real networks showed SW structure as indicated by high connectivity between neighbors and also a portion of long-range connections resembling the random network. Oscillations at the alpha frequency appeared to fulfill a pace-setting function in the real network, showing CFCs with all the frequencies used here. This strong connectivity of the alpha oscillations to all other oscillation frequencies seems to be a characteristic property of the real network constructed on the basis of CFC. Whether this network topology is characteristic for kissing only or has a broader applicability remains to be seen. Interestingly, lattice and random networks showed a comparable number of modules (4 modules in this simulation that are indicated by the different colors in Figure 2) with a low modularity value for the random network (*M* = 0.05) and a high modularity value for the lattice (*M* = 0.40). The real network was divided into 9 modules with relatively high modularity value (*M* = 0.29).

**Figure 3:**
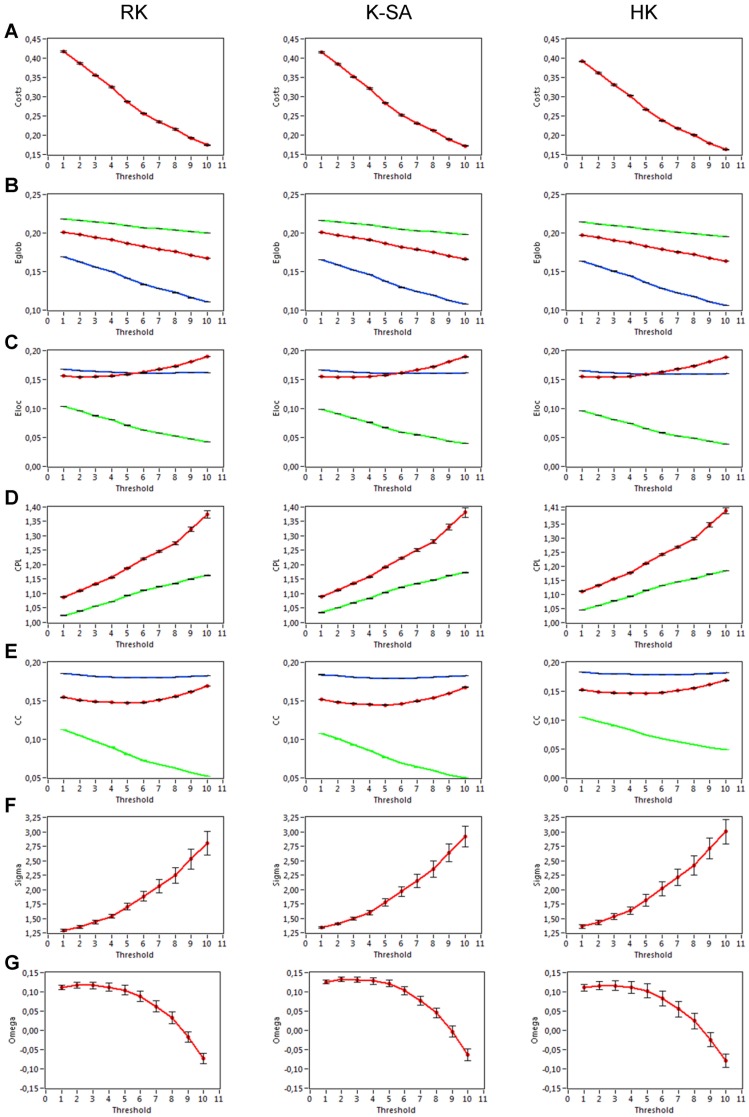
Hyper-brain network properties under the three kissing conditions. **A:** Changes in hyper-brain network costs dependent on the coupling threshold. **B:** Changes in global efficiency *(E_glob_)* in the hyper-brain, regular (lattice), and random networks dependent on the coupling threshold. **C:** Changes in local efficiency *(E_loc_)* in the hyper-brain, regular (lattice), and random networks dependent on the coupling threshold. **D:** Changes in characteristic path length *(CPL)* in the hyper-brain and random networks dependent on the coupling threshold. The *CPL* of regular networks was always equal infinity and is, therefore, not presented in the diagram. **E:** Changes in the clustering coefficient *(CC)* in the hyper-brain, regular (lattice), and random networks dependent on the coupling threshold. **F:** Changes in the small-worldness coefficient (**σ**) in the hyper-brain network dependent on the coupling threshold. **G:** Changes in the small-worldness coefficient (**ω**) in the hyper-brain network dependent on the coupling threshold. RK = romantic kissing, K-SA = kissing while performing silent arithmetic, and HK = hand kissing. Hyper-brain network: red line; regular network: blue line; and random network: green line.

The real and control networks for each couple were constructed for 10 different adaptive thresholds (see Methods for details), and the SW metrics were measured as a function of the threshold (Figure 3). As expected, increasing thresholds resulted in lower costs, which indicate sparser networks (Fig. 3A). Sparser networks have lower global but higher local efficiency (Fig. 3B and 3C), which are correspondingly related to a higher *CPL* and also a higher *CC* (Fig. 3D and 3E). Thus, sparsity in hyper-brain networks leads to higher segregation but lower integration of information flow. The small-worldness coefficient *σ* was always greater than 1 and increased with lower costs (Fig. 3F) indicating SW properties for all networks independently of the threshold or sparseness. The other small-worldness coefficient *ω* ranged between −0.3 and +0.3 for individual networks and decreased with lower costs (Fig. 3G), also indicating SW properties of the observed networks and a tendency to become more regular with higher sparseness. Further, we compared network characteristics at the eighth threshold level (f_8_ = 1.035) with high sparsity and optimal SW parameters.

**Figure 4:**
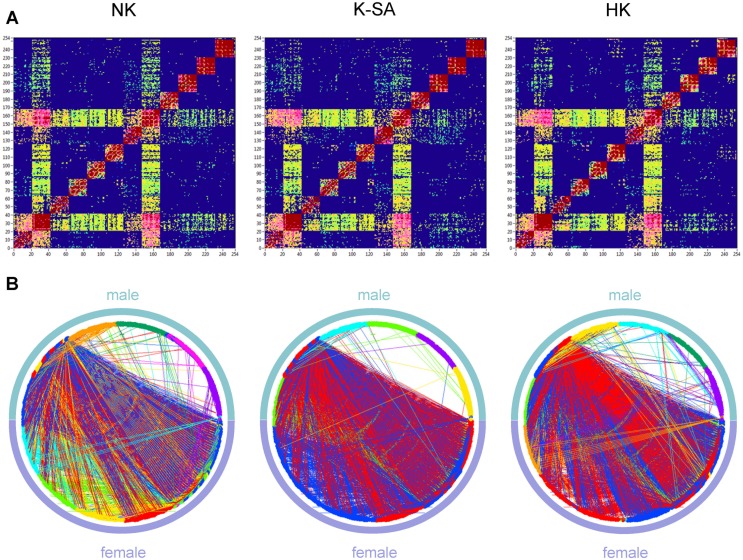
Hyper-brain network coupling matrices and circle network representations for the three kissing conditions. **A:** Coupling *(aICI)* matrices covering WFC and CFC between the 254 nodes of the kissing couple’s hyper-brain network under the three kissing conditions (RK, K-SA, and HK). **B:** The same networks as in **A**, represented in the form of a circle, where the nodes are in clockwise order. Note: The structure of the coupling matrices and the circle networks is the same as in Figures 1 and S2.

Figure 4 displays the network structures of one of the kissing couples under the three different kissing conditions. As mentioned above, alpha frequency interacting with all the other frequencies plays an exceptional role in the hyper-brain networks (Fig. 4A). Alpha frequency oscillations showed also strong CFC to theta oscillations, which remain therefore in a specific connection to each other and constitute together the so-called theta-alpha subnetwork binding the brains of the kissing couple together (Fig. 4B). This subnetwork has been observed practically in all kissing couples participating in the study. Through strong interconnection of alpha-frequency nodes with other frequencies, this subnetwork is strongly incorporated in the whole hyper-brain network structure and has a particular importance.

### Statistical Evaluation of GTA measures

#### Out-strength, Characteristic Path Length, and Clustering Coefficient

Statistical analyses carried out for the three graph-theoretical measures (out-strength, *CPL_i_*, and *CC_i_*, see Table 2) showed that: (i) strengths differed between kissing conditions, in that they were higher during RK and K-SA than during HK, (ii) *CPL_i_* also differed between kissing conditions, with the shortest path length during RK and K-SA than during HK, (iii) *CC_i_* showed only significant interaction Kissing×Site (F_4,112_ = 4.39, P<0.01) with higher clustering during RK at frontal sites and lower clustering at parietal sites. Additionally, all three measures showed significant main effects of Frequency and Site, with the strongest effect for alpha frequency (stronger coupling strength but shortest path length and lower clustering) and a predominance of coupling strength, *CC_i_* and *CPL_i_* (shortest path length) at parieto-occipital sites (s. Table 2). The kissing effect was also modulated by Frequency and Site as shown by the significant interaction Kissing×Frequency×Site for out-strength (F_20,560_ = 3.12, P<0.005, η^2^ = 0.10), with stronger strengths at 10 and 40 Hz, and at parieto-occipital sites.

**Table 2:** Statistical analysis results for network measures (*Strength*, *CPL_i_*, and *CC_i_*).

	Frequency	Site	Kissing	Posthoc Scheffetest for Kissing
*Strength*	F_5,140_ = 213.26	F_2,56_ = 29.34	F_2,56_ = 8.15	M_RK_>M_HK_
	P<0.0001	P<0.0001	P<0.005	P<0.005
	η^2^ = 0.88	η^2^ = 0.51	η^2^ = 0.23	M_K-SA_>M_HK_
				P<0.05
*CPL_i_*	F_5,140_ = 169.45	F_2,56_ = 16.25	F_2,56_ = 12.57	M_RK_<M_HK_
	P<0.0001	P<0.0001	P<0.0001	P<0.0001
	η^2^ = 0.86	η^2^ = 0.37	η^2^ = 0.31	M_K-SA_<M_HK_
				P<0.001
*CC_i_*	F_5,140_ = 57.65	F_2,56_ = 74.19	n.s.	n.s.
	P<0.0001	P<0.0001		
	η^2^ = 0.67	η^2^ = 0.73		
*Intra-brain*	F_5,140_ = 94.01	F_2,56_ = 51.25	F_2,56_ = 7.13	M_RK_>M_HK_
*strength*	P<0.0001	P<0.0001	P<0.005	P<0.005
	η^2^ = 0.77	η^2^ = 0.65	η^2^ = 0.20	M_K-SA_>M_HK_
				P<0.05
*Inter-brain*	F_5,140_ = 102.51	F_2,56_ = 11.09	F_2,56_ = 4.90	M_RK_>M_HK_
*strength*	P<0.0001	P<0.0001	P<0.05	P<0.05
	η^2^ = 0.79	η^2^ = 0.28	η^2^ = 0.15	

CPL = Characteristic Path Length, CC = Clustering Coefficient, RK = romantic kissing, K-SA = kissing while performing silent arithmetic, HK = hand kissing, M = mean, n.s. = non significant.

#### Intra- and inter-brain strengths

To test whether the strength differences between the kissing conditions were not only due to enhanced within-brain coupling, we determined strengths separately for intra- and inter-brain connections (s. Methods for details). We found a significant main effect of Kissing for both intra- and inter-brain strengths (s. Table 2). The strengths were higher during RK and K-SA than during HK for the intra-brain and higher during RK than during HK for the inter-brain coupling. The differences between kissing conditions for inter-brain coupling were modulated by electrode site, F_4.112_ = 3.62, P<0.05, η^2^ = 0.12, with stronger differences between kissing conditions at parietal sites. We also found significant main effects of Frequency and Site, with stronger coupling strength at alpha frequency and at parieto-occipital sites.

Additionally, we tested the strength of lip EMG channels using a two-way repeated-measures ANOVA (Sex×Kissing). There were no significant differences between sex groups or kissing conditions.

#### Correlation between GTA measures and subjectively assessed kissing satisfaction

**Figure 5:**
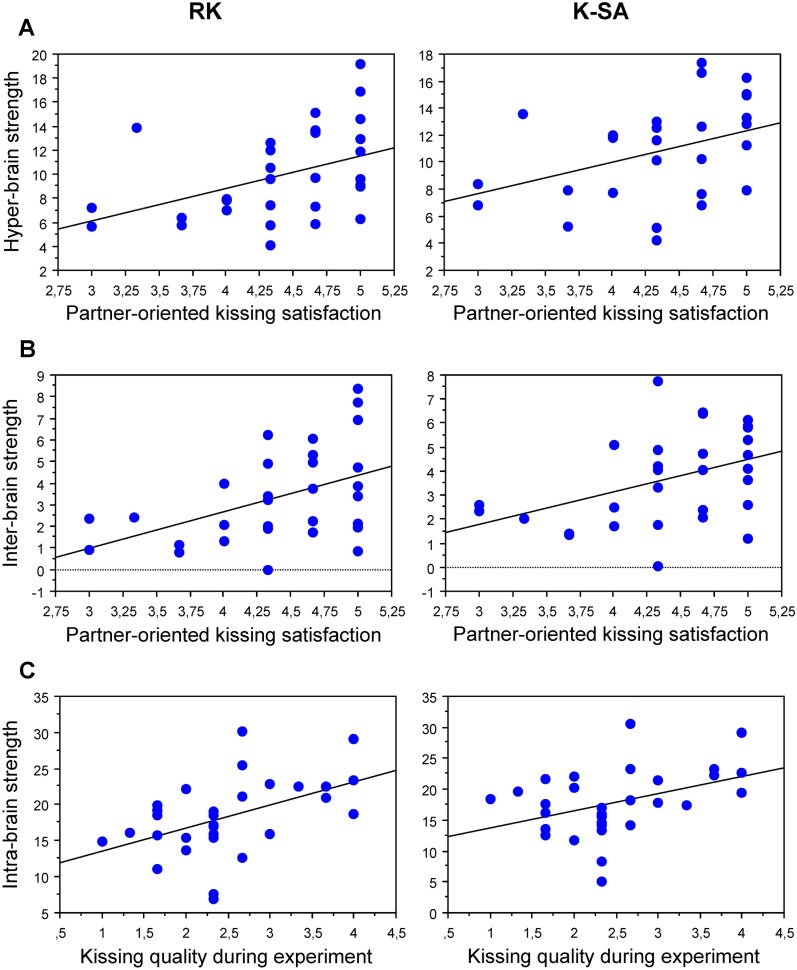
Correlation plots displaying the correlation between strengths (hyper-, intra-, and inter-brain) and subjectively assessed partner-oriented kissing satisfaction and immediate kissing quality. **A:** Correlation between the hyper-brain strength (5-Hz oscillation nodes) and partner-oriented kissing satisfaction under the two kissing conditions (RK and K-SA). **B:** Correlation between the inter-brain strength (5-Hz oscillation nodes) and partner-oriented kissing satisfaction under the two kissing conditions (RK and K-SA). **C:** Correlation between the intra-brain strength (10-Hz oscillation nodes) and kissing quality (assessed after K-SA) under the two kissing conditions (RK and K-SA).

Strength and *CPL_i_* for theta-oscillation nodes (5 Hz) in the hyper-brain network and especially for inter-brain (but not for intra-brain) connections during RK and K-SA conditions correlated significantly with items of partner-oriented kissing satisfaction (see Table 3 and Figure 5). Correlations during HK did not differ reliably from zero, and the correlation between partner-oriented kissing satisfaction and inter-brain strength during RK was significantly different from the corresponding correlation during HK (Zd = 1,97, P<0.05). Intra-brain strength for alpha-oscillation nodes (10 Hz) during RK and K-SA correlated significant positively with immediate kissing quality assessed after K-SA. Furthermore, there were significant correlations between kissing quality assessed after K-SA and hyper-brain strength (positive) as well as *CPL_i_* (negative) during RK. Unexpectedly, kissing quality assessed after RK was negatively correlated to hyper-brain strength and *CPL_i_* during RK and to parieto-occipital hyper-brain strength during K-SA.

**Table 3:** Correlation coefficients for strength (hyper-, Intra-, and inter-brain) and characteristic path length (*CPL_i_*) with average scores of subjective partner-oriented kissing satisfaction and immediate kissing quality during the task.

	Kissing satisfaction			Kissing quality			
				Assessed during RK		Assessed during K-SA	
	RK	K-SA	HK	RK	K-SA	RK	K-SA
	Strengths (hyper-brain connections)						
Frontal	0.452*	0.390*	0.297	−0.382*	−0.297	0.424*	0.342
Central	0.342	0.278	0.271	−0.377*	−0.290	0.359	0.301
Parietal	0.316	0.319	0.213	−0.419*	−0.368*	0.356	0.173
	Strengths (intra-brain connections)						
Frontal	0.324	0.210	0.226	−0.219	−0.066	0.479**	0.409*
Central	0.188	0.154	0.217	−0.201	−0.090	0.414*	0.388*
Parietal	0.210	0.169	0.204	−0.257	–0.133	0.444*	0.308
	Strengths (inter-brain connections)						
Frontal	0.461**	0.427*	0.302	–0.333	–0.305	0.162	0.049
Central	0.420*	0.302	0.256	–0.324	–0.287	0.129	0.037
Parietal	0.363*	0.390*	0.191	–0.355	–0.350	0.124	–.057
	Characteristic Path Length (*CPL_i_*)						
Frontal	–0.360*	–0.309	–0.293	0.385*	0.265	–0.398*	–0.311
Central	–0.329	–0.218	–0.226	0.341	0.255	–0.309	–0.261
Parietal	–0.267	–0.250	–0.211	0.413*	0.347	–0.323	–0.191

RK = romantic kissing, K-SA = kissing while performing silent arithmetic, and HK = hand kissing.

Please note that partner-oriented kissing satisfaction was correlated with theta oscillation nodes (5 Hz) and kissing quality was correlated with alpha oscillation nodes (10 Hz).

### Modularity structures during kissing

Next, we investigated modularity structures of the 15 individual hyper-brain networks, which could be partitioned well into different modules or communities, with modularity values ranging between 0.24 and 0.47 (M = 0.32, SD = 0.06). Modularity values were reliably higher than values observed in random networks (p<0.0001) indicating the presence of nonrandom community structures. The number of modules varied between 4 and 14 with an average of 8.4 (SD = 2.2) modules for all individual hyper-brain networks and kissing conditions. There were no significant differences in the number of modules and modularity between kissing conditions.

**Figure 6:**
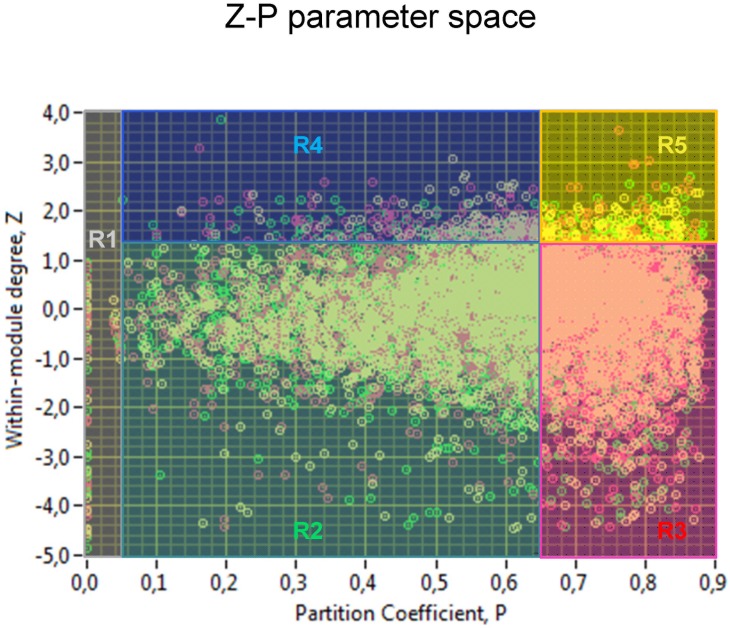
*Z-P* parameter space with corresponding roles for all kissing couples. Scatterplot of *Z-P* parameter space characterized by the participation coefficient *P* (X-axis) and the within-module degree *Z* (Y-axis) representing all kissing couples under the three kissing conditions. Roles with corresponding regions: R1 (*P_i_*≤0.05) – ultra-peripheral nodes; R2 (*Z_i_*<1.4; 0.05<*P_i_*<0.65) – non-hub peripheral nodes; R3 (*Z_i_*<1.4; 0.65<*P_i_*<0.9) – non-hub connector nodes; R4 (*Z_i_*>1.4; 0.05<*P_i_*<0.65) – hub peripheral nodes; R5 (*Z_i_*>1.4; 0.65<*P_i_*<0.9) – hub connector nodes. RK: yellow circles; K-SA: red circles; and HK: green circles.

To define how a node was positioned within its own module and with respect to other modules, we calculated the within-module degree (*Z_i_*) and participation coefficient (*P_i_*) of the node *i* for the hyper-brain networks of the kissing couples. The within-module degree measures how ‘well-connected’ node *i* is to other nodes in the module, whereas participation coefficient reflects how ‘well-distributed’ the links of the node *i* are among the other modules. Together, *Z_i_* and *P_i_* form the so-called *Z-P* parameter space, with different regions indicating specific universal roles of the nodes positioned in this space or these regions. Although some attempts to define these regions or roles were reported in the literature [54]–[57], in our opinion, the boundaries of the roles in the given Z-P parameter space can not always be identical when using different measures and having different network structures. Therefore, they should be determined individually based on some specific rules. Figure 6 displays the Z-P parameter space for the three kissing conditions including all kissing couples. It can be seen that the topology of the Z-P parameter space is symmetric to the horizontal axis at *Z_i_* = 0 and have a specific bulb-like form. Nodes with the within-module degree *Z_i_*≥1.4 were defined as hubs and nodes with *Z_i_*<1.4 as non-hubs. We adopt this separation Z-value from our previous study with guitarists [8], which corresponds to the definition of hubs as nodes containing many more edges than most of the nodes in the module (cf. [54]). The number of hubs in our hyper-brain networks calculated across all kissing couples in the three kissing conditions was 4.8%. The boundary for provincial and connector nodes was set to 0.65, separating 48.6% of connector nodes. This separation value for provincial and connector nodes is close to the value suggested by Guimerà and Amaral [54]. In addition, we distinguished ultra-peripheral nodes (P≤0.05) and kinless nodes (0.9≤P≤1.0). Corresponding to this separation, the *Z-P* parameter space was divided into six different roles:

R1 (*P_i_*≤0.05) – ultra-peripheral nodes,
R2 (*Z_i_*<1.4; 0.05<*P_i_*<0.65) – non-hub peripheral nodes,
R3 (*Z_i_*<1.4; 0.65<*P_i_*<0.9) – non-hub connector nodes,
R4 (*Z_i_*>1.4; 0.05<*P_i_*<0.65) – hub peripheral nodes,
R5 (*Z_i_*>1.4; 0.65<*P_i_*<0.9) – hub connector nodes,
R6 (*P_i_*>0.9) – kinless nodes.

**Figure 7:**
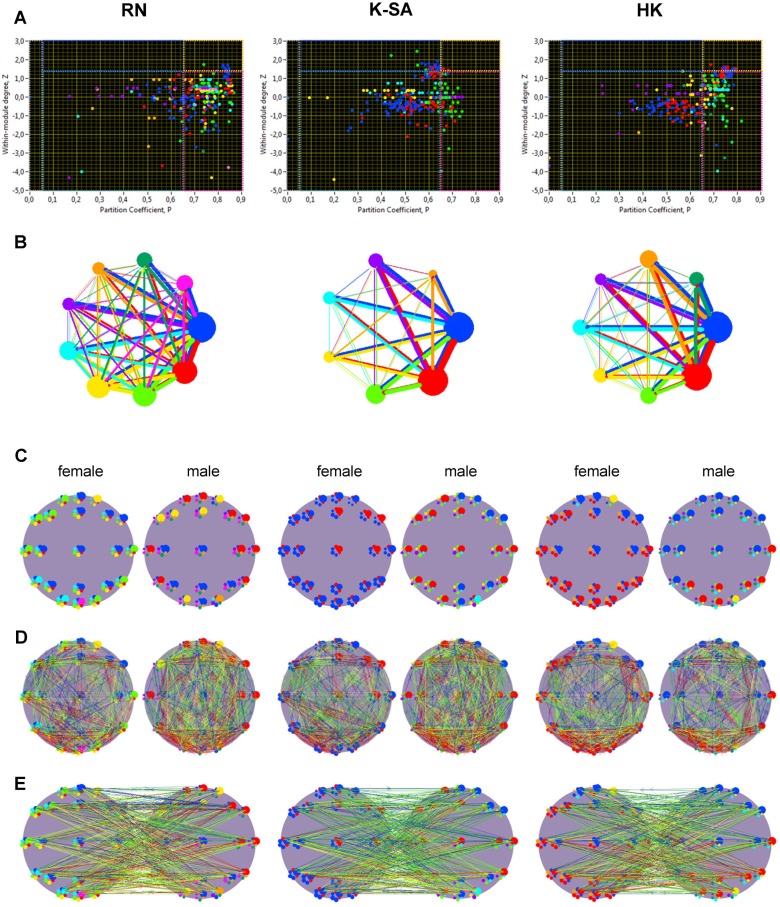
Modular organization of hyper-brain networks under the three kissing conditions. **A:** Scatterplots of *Z-P* parameter space with corresponding role regions (cf. the. 5). Different modules are coded with color of the circles. **B:** Circle modularity structure. The size of the circle (module) represents the common connectivity strength of the module, and connectivity strength between the modules is coded by line thickness. In both cases, the out-strengths were used for calculation. **C:** Modular organization of the female and the male brains. Each electrode contains six nodes representing different oscillation frequencies (5, 10, 20, 30, 40, and 60 Hz) in clockwise order, beginning from the top. The size of the circle corresponds to the out-strength of the node, and modules are represented by color, which is the same as in **A** and **B**. **D:** Within-brain connections in the female and the male brains, correspondingly. Coupling strength range from blue (low coupling) to red (high coupling). **E:** Between-brain connections between the female and the male brains. Coupling strength range from blue (low coupling) to red (high coupling).

Figure 7 displays the structure of the *Z-P* parameter space of a kissing couple under the three kissing conditions, with the nodes belonging to different modules coded by colors. It can be seen that most of the connector and also peripheral hubs share the same two or three largest modules in the network (Fig. 7A). In most cases of the kissing couples investigated in the study, the largest module represents the aforementioned theta-alpha subnetwork. This module also has the strongest connections to the other modules (Fig. 7B). However, a more thorough view on the modularity structure of the kissing couple presented in Figure 7 indicates that the modular organization of hyper-brain networks have a more complex structure (see Fig. 7C). During RK, five out of nine (in total) modules (marked in blue, red, green, yellow, and aquamarine) are relatively large and contain alpha-oscillation nodes together with other frequency nodes distributed across the two brains. The blue module shares theta- and alpha-frequency nodes in the two brains and represents the above-mentioned theta-alpha subnetwork. Furthermore, fronto-temporal alpha-frequency nodes in male brain share the beta-frequency nodes distributed across the female brain in the common module marked in red. The yellow module shares the frontal (and one occipital) alpha-frequency nodes in the male brain with 30-Hz-oscillation nodes distributed across the female brain. The green and aquamarine modules belong practically to the female brain and bind together alpha and gamma oscillations in the female brain. All this leads to a very intertwined hyper-brain structure during RK. During K-SA, there are two (marked in blue and red) out of seven (in total) largest modules, whereby all the nodes in the female brain belong to these two largest modules and share these modules with alpha-frequency nodes in the male brain. Overall, these two modules represent two very strong hyper-brain subnetworks: (i) alpha-gamma subnetwork (blue) and (ii) theta-alpha-beta subnetwork (red). During HK, there are two large modules (marked in blue and red) that share alpha-frequency nodes in the male brain with those of the female brain, whereby all the frequencies, with exception of 60-Hz oscillations in the female brain, join in these two modules; moreover, the nodes belonging to the first largest module (marked in blue) lie in the fronto-central regions in both the female and male brains, whereas the nodes from the second largest module (marked in red) are localized in the temporal and parieto-occipital regions, also in both brains. It thus appears that hyper-brain networks in kissing couples have a complex modular organization, in which alpha-frequency oscillations and their subnetworks, especially the theta-alpha subnetwork, play a crucial role. Figures 7D and 7E show the strongest connections within and between a couple’s brains, respectively. It should be noted here that intra-brain networks based on CFC have very strong large-scale connections binding distributed cell assemblies, especially between the frontal and parieto-occipital areas. These regions are also strongly interconnected or synchronized between the brains. Inter-brain coupling generally reach out from the frontal regions of one partner to the parieto-occipital or central regions of the other partner and vice versa, whereas frontal-to-frontal connections are mostly reduced or attenuated.

Further, we calculated the numbers of nodes lying in the different regions of the Z-P parameter space for women and men separately under the three kissing conditions. (see Table 4). A two-way repeated measures ANOVA (Kissing×Sex) showed no significant differences between kissing conditions or sex groups at all. There is a tendency for a higher number of hubs and non-hub connectors and a smaller number of non-hub peripheral nodes during RK, compared with control conditions, but these differences were not statistically reliable.

**Table 4:** Number of nodes (mean and standard deviation in %) lying in the specific regions of the *Z-P* parameter space separately for women and men under the three kissing conditions.

Roles	female			male		
	RK	K-SA	HK	RK	K-SA	HK
R1	0.9 (1.7)	1.2 (2.2)	0.9 (2.1)	0.5 (0.5)	0.3 (0.4)	0.8 (1.1)
R2	47.3 (24.1)	49.9 (27.4)	50.7 (16.3)	44.4 (20.0)	46.2 (25.9)	48.9 (19.1)
R3	46.8 (25.0)	44.3 (24.3)	44.0 (18.6)	50.0 (22.9)	47.9 (28.1)	46.2 (21.2)
R4	2.4 (3.4)	3.3 (3.6)	1.9 (2.3)	3.6 (4.1)	3.3 (4.4)	2.0 (2.6)
R5	2.7 (3.3)	1.4 (1.9)	2.5 (2.8)	1.5 (2.2)	2.3 (3.0)	2.0 (2.9)

R1 = ultra-peripheral nodes, R2 = non-hub peripheral nodes, R3 = non-hub connector nodes, R4 = hub peripheral nodes, R5 = hub connector nodes, RK = romantic kissing, K-SA = kissing while performing silent arithmetic, and HK = hand kissing. Note that there were no kinless nodes (R6) at all.

## Discussion

In this article, we reported a network analysis based on graph theory to examine cross-frequency coupling within and between the brains of kissing couples. Our key finding is that CFC hyper-brain networks consisting of the two brains of the interacting people have SW properties, and that alpha frequency oscillations play an essential role in these networks, as they set the pace for other frequencies in the network. Furthermore, we identified theta-alpha subnetworks that bind together the two brains of kissing partners, especially during partner-oriented kissing, and that play a crucial role during interpersonal action coordination. Our analyses introduce a new method for capturing CFC in hyper-brain networks.

### Advantages of a network architecture based on cross-frequency coupling

The human brain is a complex system that shows temporally coherent activity at multiple scales of time and space. This activity constitutes functional networks of different topology intermediate between highly regular lattices and random graphs. This topology is called SW topology [45]. We have found that our complex hyper-brain networks based on CFC within and between the brains of interacting people also possess SW topology, with the high degree of clustering found in a lattice and the short path length found in a random graph. We investigated our real and control networks for each participating couple by using 10 different adaptive thresholds corresponding to different sparsity levels that increase by threshold. Sparser networks showed higher local efficiency but lower global efficiency, which are related to higher *CC* and also higher *CPL*. Despite the fact that sparsity in hyper-brain networks led to higher segregation, but also to lower integration of information flow, the small-worldness coefficient *σ* increased with lower costs and was always greater than 1, indicating that real networks retained small-world network topology at all threshold levels. Moreover, the other small-worldness coefficient *ω* ranged between −0.3 and +0.3, also indicating that the observed networks belong to SWNs. About half of the kissing couples showed more random properties (*ω*>0), whereas the other half showed a more regular network properties (*ω*<0). A decrease of the coefficient *ω* with a higher threshold or lower costs indicates a tendency of networks to become more regular. For further analyses, we chose the threshold level that provided a high sparsity of networks with about equal local and global efficiency. Modularity analyses showed that hyper-brain networks at this threshold level had mean modularity values at about 0.3, which were statistically always higher than that in random networks. This indicates nonrandom community structure in hyper-brain networks [56]. Furthermore, community structures were organized by combining electrode location and oscillation frequency, whereby each electrode oscillating at different frequencies participated in multiple modules. This gives rise to overlapping community structures within and between the brains. Normally, low-frequency nodes (e.g., theta and alpha) were distributed across the two brains and composed the so-called hyper-brain *modules* sharing electrodes/nodes from two brains (c.f. [8], [9]). The largest hyper-brain module was normally the theta–alpha subnetwork, which could be found in practically all kissing couples. Besides this largest hyper-brain module, there were also smaller ones composed from other also high-frequency nodes. Such hyper-brain modules were also found in our earlier studies based on within-frequency analyses [8], [9]. Hyper-brain networks based on CFC, however, have the advantage that they show how different frequencies interact with each other and build up overlapping community structures. This allows a better understanding of network topology and its organizational principles [58], [59]. Neural cell assemblies are normally organized on the principle of overlapping communities, with neural units being shared by different overlapping cell assemblies [34]–[37]. The networks observed here follow the same principles and are therefore suitable for the investigation of hyper-brain networks arising during interpersonal action coordination.

### Kissing satisfaction, lip EMG and EEG alpha activity

In terms of subjective experience, we found that women and men did not differ regarding partner-oriented kissing satisfaction but that women generally reported higher immediate kissing quality during the experiment than men. Not surprisingly, kissing quality was rated higher during RK than during K-SA. Women and men did not differ in peak alpha frequency. However, alpha peak frequency was higher during K-SA than during RK or HK, which may reflect the cognitive demand of executing both tasks (kissing and silent arithmetic) simultaneously. Additionally, spectral power in the low and high alpha frequency bands varied as a function of the kissing conditions and as a function of sex. The spectral power was generally highest during RK and lowest during K-SA in the low alpha frequency band, and lowest during HK in the high alpha frequency band, especially at parieto-occipital regions. This means that RK enhances spectral power compared with HK in both low and high frequency bands, whereas K-SA is associated with higher spectral power in the high frequency band only. Likewise, men showed enhanced spectral power in the low alpha frequency band, especially at parieto-occipital sites, whereas women showed enhanced power in the high alpha frequency band, specifically at occipital and frontal regions. Despite this variation of alpha spectral characteristics by the sex and kissing conditions, neither alpha peak frequency nor alpha spectral power correlated significantly with kissing satisfaction or quality.

We also determined the average power of lip EMG across the individual maximum (±10 Hz) and at 60 Hz (±10 Hz), and the latter was used together with EEG channels for the construction of hyper-brain networks. Women showed generally higher lip EMG power than men, indicating stronger lip activity during kissing, independently of kissing condition. Lip activity also did not show any reliable correlations with kissing satisfaction or immediate quality of kissing during the experiment. It indicates that neither alpha band activity nor lip muscle activity are responsible for kissing satisfaction, at least not responsible alone.

### Kissing from the viewpoint of network properties

Network analyses showed that hyper-brain networks during partner-oriented kissing (especially during RK) as compared with hand kissing have elevated strength and shortest path length indicating higher connectivity between different brain regions within and between the brains, and also optimized information transfer within and between them. The fact that higher strength during partner-oriented kissing was found for both intra- and inter-brain connections indicates that neural cell assemblies synchronize stronger not only within the brains of kissing partners but also between their brains. Thus, this evolutionarily important activity is associated with enhanced synchronicity between the brains of kissing partners. Such elevated inter- and also intra-brain connectivity was previously found with guitar duets [7]–[10], during spontaneous imitation of hand movements [19], [60], and during card playing [18], [20]. In these studies, cross-frequency coupling was not considered, and there was a preference for low (delta, theta) communication frequencies (cf. [7]–[9]) to bind two interacting brains. In other studies, especially when synchronization was measured across time [8], [9], [18]–[20], higher frequencies (e.g., alpha, beta, and gamma) were involved as well. The important role of alpha observed in this study, however, has not been observed previously. Our results suggest that the important role of alpha is due to CFC, in the sense that the alpha frequency sets the pace for other frequencies. This interpretation is consistent with the available evidence. Alpha oscillations serve an important function in top-down attentional processes [61]–[63], and they synchronize with oscillations at other frequencies in response to cognitive demands [59], [62], [64], [65]. The theta-alpha subnetwork identified in this study is in good agreement with these earlier results. This subnetwork comprises nearly all electrode sites in the two brains oscillating at theta or alpha frequencies, whereby the electrodes oscillating at alpha frequency (mostly at parieto-occipital sites) fulfill the role of a connector hub, with strong connections to the other nodes of the subnetwork as well as to nodes or electrodes belonging to other modules or subnetworks. Based on these observations, we tentatively conclude that parieto-occipital sites in the kissing partners may have played a leading or integrating role during kissing.

Kissing differs from other types of social interaction, such as music or card playing, by stronger reciprocal sensory and motor connections, including afferent and efferent feedback loops. Additionally to EEG couplings, we observed significant coupling between the lip EMGs of the two partners (lip–lip), as well as couplings between lip EMG and EEG within and across interaction partners. Within each partner, the EEG oscillations with the same frequency as the lip EMG (i.e., 60 Hz) were mostly strongly connected with the lip EMG. On the other hand, alpha frequency EEG oscillations were also strongly coupled, both with the own lip EMG and the lip EMG of the kissing partner. Couplings between EMG and EEG have been reported previously [66]–[68], but, for the first time, we show that EMG activity is synchronized with EMG and EEG activity of the interacting partner.

A further finding of this study is the significant correlation between strength (and also *CPL_i_*) and subjectively assessed kissing satisfaction and quality. The coupling strength and integrative capacity of the frontal nodes and also of other brain regions may underlie this association. We only tested 5- and 10-Hz oscillations, which play an important role in the between-brain connectivity binding two brains together mostly in terms of theta-alpha subnetworks distributed across the two brains of kissing partners. Frontal theta hyper-brain strength during RK and K-SA and *CPL_i_* during RK showed reliable relationship to partner-oriented kissing satisfaction. Importantly, this relationship was strongest for inter-brain connections covering all brain regions during RK, and frontal and parietal regions during K-SA. Given the fact that intra-brain strengths did not show a reliable relationship, it can be concluded that partner-oriented kissing satisfaction is associated with distributed inter-brain connectivity and frontal nodes in hyper-brain networks, showing strong connectivity and integration processes (shortest path length) in common hyper-brain network. Given the relatively low spatial resolution of EEG, we can only speculate about the neural circuitry below the frontal electrodes that form the substrate for this hyper-brain partner-oriented kissing-satisfaction effect. Neural activity in the medial prefrontal cortex is selectively enhanced during theory-of-mind tasks and mentalization [69]–[71]. Strong involvement of frontal regions in the within-brain, and especially the between-brain, synchronization has also been found with guitar duets [7]–[9]. Thus, these coupling strengths may reflect a synchronization of cell assemblies representing the coordination of one’s own behavior with the behavior of the interaction partner [2], [3], [17], [72]. This line of reasoning is further supported by the observation that kissing satisfaction correlated reliably with inter-brain but not with intra-brain strength. Thus, the significant relationship of kissing satisfaction with strength and *CPL_i_* during partner-oriented kissing (RK and K-SA) but not during hand kissing suggests that orientation toward the partner during kissing not only enhances frontal hyper-brain circuitry and distributed inter-brain coupling strength, but also induces neural integration (reduced *CPL_i_*) in the whole hyper-brain network. This, in turn, may enhance the coordination of kissing behavior and enhance kissing satisfaction.

Surprisingly, the immediate effect of kissing quality during the experiment showed a different relationship with network indicators dependent on whether kissing quality was assessed after the RK or K-SA test session. Kissing quality assessed after K-SA showed a positive association with hyper-brain strength and a negative association with *CPL_i_* for 10-Hz oscillation nodes during RK, whereas kissing quality assessed after RK showed an inverse relationship with hyper-brain strength and *CPL_i_* during RK, and also with parieto-occipital hyper-brain strength during K-SA. At the same time, intra-brain strength for alpha-oscillation nodes during RK and K-SA correlated positively with immediate kissing quality assessed after K-SA. This relationship with kissing quality assessed after RK was not reliable and rather negative. It seems that the assessment of kissing quality after a K-SA session is more veridical than after RK. Most of the participants reported after this session (K-SA) that combining kissing with performing silent arithmetic has been circumstantial, and they probably paid more attention to kissing and assessed them more differentially.

Kissing quality correlated reliably with intra-brain strength, but not with inter-brain strength, whereas the relationship to partner-oriented kissing satisfaction was inverse, that is, it correlated reliably with inter-brain, but not with intra-brain strength. It seems that kissing quality is related above all to within-brain dynamics, whereas partner-oriented kissing satisfaction addresses above all the dynamics between the brains. Moreover, the former is related to alpha oscillations, whereas the latter to theta oscillations, which are slower. In a previous study [8], we also found that the inter-brain connectivity was operating at lower frequencies than intra-brain connectivity. It remains to be seen whether these observations form part of a more general phenomenon.

## Limitations

The present experiment has limitations and leaves room for questions to be addressed in future research. First, we used only six frequencies of interest for network construction. Using more fine-grained frequency components would lead to a more differentiated representation of hyper-brain networks. At the same time, our results clearly show the benefits of including CFC in network construction. Second, our analyses were limited to phase-to-phase CFC. Other types of CFC (e.g., power to power, phase to power, power to frequency, or phase to frequency) are likely to provide further information about functionally relevant network properties [22]. Third, the coupling measures used in this study were linear and bivariate. Nonlinear and multivariate couplings [73], [74] may also contribute to inter-brain dynamics and should be investigated in the future. In conjunction with high-density behavioral assessments, such measures may shed further light on the behavioral and neuronal dynamics of interpersonal action coordination. Finally, the order of RK and K-SA was fixed instead of counterbalanced to safeguard the ecological validity of RK. Hence, an unknown portion of the effects attributed to condition may have been due to sequence effects.

## Conclusion

Methodologically, the results of this study show that hyper-brain networks constructed on the basis of CFC have clear advantages in the investigation of neural mechanisms of interpersonal action coordination. Such networks consider the interactions of different frequencies and incorporate overlapping community structures, and thereby provide a more complete representation of network topology and organization. We showed that CFC-based hyper-brain network topology differ between partner-oriented and solitary kissing. Significant relations of network properties, such as strength and average shortest path length (*CPL_i_*), to subjectively assessed partner-oriented kissing satisfaction, point to the functional significance of hyper-brain network properties for interpersonal action coordination. Finally, this study shows that data acquisition and analysis methods for simultaneous EEG and EMG recordings from multiple persons are important tools to examine inter-brain oscillatory couplings during interpersonal interactions.

## Supporting Information

Figure S1 **Construction of the hyper-brain network using a CFC approach. A:** Coupling *(aICI)* matrix covering within frequency coupling (WFC) and cross frequency coupling (CFC) between the 254 nodes of the kissing couple’s hyper-brain network. The nodes are organized by electrode location (Fp1, Fpz, Fp2, F7, F3, …, O2), oscillation frequency (5, 10, 20, 30, 40, and 60 Hz), and brain (female, male); the last two nodes are lip EMG channels oscillating at 60 Hz for a female and a male, correspondingly. **B:** The same network as in **A**, represented in the form of a circle, where the nodes are in clockwise order for the female and the male nodes, representing 21 electrodes for each of the six frequencies used for network construction. The last two nodes are the lip EMG channels. It can be seen that most of the long-range connections are between the theta (5 Hz) and alpha (10 Hz) frequency nodes of the female and the male brains, representing the so-called theta-alpha subnetwork.
(TIF)

Table S1 **Psychological assessment of partner- and relationship-oriented satisfaction.**
(DOCX)
