# Supplementary figures and images for: Hyper-Brain Networks Support Romantic Kissing in Humans
Source: PLoS One. 2014 Nov 6;9(11):e112080. doi: 10.1371/journal.pone.0112080 (PMC4222975; doi:10.1371/journal.pone.0112080)

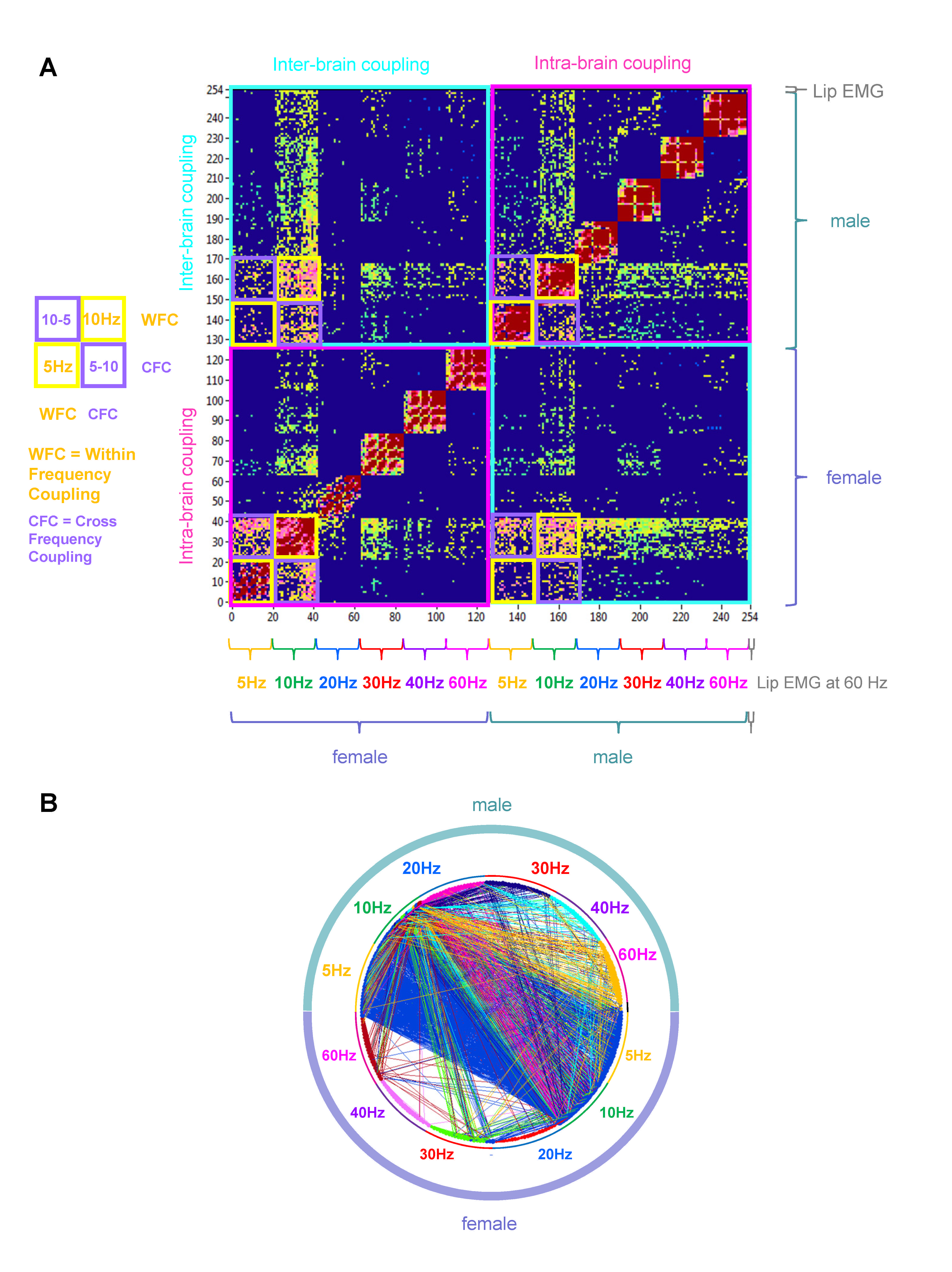

Supplement: Figure S1 — Construction of the hyper-brain network using a CFC approach. A: Coupling (aICI) matrix covering within frequency coupling (WFC) and cross frequency coupling (CFC) between the 254 nodes of the kissing couple’s hyper-brain network. The nodes are organized by electrode location (Fp1, Fpz, Fp2, F7, F3, …, O2), oscillation frequency (5, 10, 20, 30, 40, and 60 Hz), and brain (female, male); the last two nodes are lip EMG channels oscillating at 60 Hz for a female and a male, correspondingly. B: The same network as in A, represented in the form of a circle, where the nodes are in clockwise order for the female and the male nodes, representing 21 electrodes for each of the six frequencies used for network construction. The last two nodes are the lip EMG channels. It can be seen that most of the long-range connections are between the theta (5 Hz) and alpha (10 Hz) frequency nodes of the female and the male brains, representing the so-called theta-alpha subnetwork. (TIF) [file pone.0112080.s001.tif]
