# Supplementary material for: Hyper-Brain Networks Support Romantic Kissing in Humans
Source: PLoS One. 2014 Nov 6;9(11):e112080. doi: 10.1371/journal.pone.0112080 (PMC4222975; doi:10.1371/journal.pone.0112080)
Supplement: Table S1 — Psychological assessment of partner- and relationship-oriented satisfaction. (DOCX) [file pone.0112080.s002.docx]

**Table S1.** Psychological assessment of partner- and relationship-oriented satisfaction

| Items | Female | | Male | |
| --- | --- | --- | --- | --- |
| **Partner-oriented items (duration in months)** | | | | |
| Since when have you been in love? | 7.5 (4.2) | | 7.7 (4.7) | |
| How long have you been a couple? | 6.9 (3.5) | | 6.9 (3.2) | |
| How long have you known each other independent of the duration of your relationship? | 20.9 (15.3) | | 21.3 (14.6) | |
| **Relationship-oriented satisfaction** | | | | |
| How well does your partner fulfill your wishes and needs? | 4.6 (0.5) | | 4.3 (0.5) | |
| How satisfied are you with your relationship in general? | 4.6 (0.5) | | 4.5 (0.6) | |
| How good is your relationship, compared to most other couples? | 4.5 (0.6) | | 4.7 (0.5) | |
| How often do you wish that you would rather not have this relationship? | 1.1 (0.4) | | 1.4 (0.6) | |
| How well does your relationship fulfill your initial expectations? | 4.4 (0.6) | | 4.5 (0.6) | |
| How much do you love your partner? | 4.9 (0.3) | | 4.7 (0.6) | |
| How many problems are there in your relationship? | 2.5 (0.9) | | 2.1 (0.8) | |
|  |  | |  | |
| **Immediate kissing quality during experiment (“yes” responses in %)** | | | | |
|  | RK | K-SA | RK | K-SA |
| Were you sexually aroused when kissing during the experiment? | 73.3 | 26.7 | 46.7 | 20 |
| Did you also kiss with your tongue during the experiment? | 100 | 93.3 | 100 | 86.7 |

RK = romantic kissing, K-SA = Kissing while performing arithmetic task
